# The Thirty-Fourth Long Fox Memorial Lecture: Plant Hormones

**Published:** 1946

**Authors:** M. Skene

**Affiliations:** Melville Wills Professor of Botany in the University of Bristol


### The Bristol Medico-Chirurgical Journal

*" Scire est nescire, nisi id me*
*Scire alius sciret."*

WINTER, 1946.

### THE THIRTY-FOURTH LONG FOX MEMORIAL LECTURE:

BY

PROFESSOR M. SKENE, D.Sc.,

*Melville Wills Professor of Botany in the University of Bristol.*

DELIVERED IN THE UNIVERSITY OF BRISTOL ON TUESDAY, DECEMBER 11th, 1945,

ON

### PLANT HORMONES

### SUBSTANCES WHICH CONTROL PLANT GROWTH

PLANT hormones are a group of organic materials, produced by the plant itself, and affecting its growth in a variety of ways. These substances have many similarities with the familiar hormones of the animal kingdom. Although it was suspected nearly a century ago that certain regulatory effects in the plant might be due to such substances, real knowledge of their nature and activities has been achieved only in the last twenty years. It came in an indirect way.

The plant is a sedentary organism ; but its organs carry out a great variety of movements which are essential to its proper functioning. Of these the most important are the tropisms, or directed movements, set going by an external stimulus and taking place in a direction related to that in which the stimulus reaches the plant. An example is geotropism, the response to gravity which brings the main root vertically downwards in the soil and the main shoot vertically upwards in the air. Another example is phototropism, the response to unilateral illumination. The shoot grows towards the light, the leaf becomes so oriented that its blade is normal to the incident radiation. To say that the plant *grows* towards the light is literally true, for the most characteristic mechanism of plant movement is a differential growth rate on the two sides of an organ: the upper side of a root grows faster than the lower and the root bends downwards. Such movements are necessarily slow in comparison with the muscular movements of an animal; often we note that they have taken place only after the lapse of days. Yet, with suitably sensitive methods of observation, the fact that a movement is in progress may often be demonstrated a few minutes after its start.

The phototropic movement is important for our subject and we must follow it further. It was first shown by Darwin that, in a reacting seedling, the region most sensitive in the perception of the stimulus of light is the extreme tip, while the reaction by bending takes place some distance back. If the tip is cut off, or merely shaded, there is no reaction or a very limited one. The response is therefore not a direct one. It might be said that the tip acts as an organ of special sense, that there is a conduction of excitation and that the more basal parts form the motor organ. The analogy with a reflex movement in an animal is striking but superficial. The plant possesses no nervous system. How is the stimulus perceived and how is the excitation conducted?

The attack on this problem succeeded because a highly suitable laboratory object was available in the seedling of the oat. The first leaf of this seedling is a white, cylindrical sheath, called the coleoptile. It is extremely sensitive to light and its phototropic movement is considerable after an hour or so. Moreover the seedlings can be easily raised in large numbers showing uniform behaviour. If the tip is shaded the coleoptile does not react. If 1 mm. of the tip is cut off, the coleoptile fails to react and also ceases to grow. If the tip is replaced the coleoptile grows once more and also reacts to light. The simplest explanation of this behaviour is that some substance is produced in the tip and diffuses downwards and that this substance is necessary for growth. That this is so was first definitely shown by the young Dutch botanist, F. W. Went,[1] in 1928. He was not, at that time, able to extract the substance with water or other solvents, but he devised another method by which its properties could be studied. He cut off coleoptile tips and placed them on thin sheets of agar jelly for an hour or two. The agar was then cut into little cubes. If one of these was placed on the stump of a decapitated coleoptile the stump started to grow once more. This is decisive proof that we are dealing, not with anything of the nature of a nervous impulse, but with a substance which diffuses first into the agar and from that into the seedling where it has its effect on growth. The substance was named by Went "auxin," and this term has since been used for the class of substances which we now know to affect plant growth.

Even more important than this demonstration is the fact that the method enables us to measure the amount of auxin and thus to bring quantitative methods into operation. Within limits the amount of growth is proportional to the amount of auxin. It is not practicable to measure the increase of straight growth. But, if one of the agar cubes is placed at one side of the coleoptile stump, the part below it grows faster and the coleoptile bends. The angle of bending may be readily and exactly measured and is a measure of the growth effect and so of the amount of auxin present. The technique is delicate and must be carried out under strictly controlled conditions, but, as a routine, is reliable and accurate. Went was able to show that auxin is an organic substance readily destroyed by alkalies and with a molecular weight of about 370. He also demonstrated the remarkable fact that its transport in the plant is polar—it moves only from tip to base and not in the reverse direction. It was shown that auxin is produced in the tips of many—and presumably all—higher plants, especially in the embryonic leaves, and that the auxin of different plants is probably the same substance.

Auxin could not be extracted from the coleoptile, partly because the amounts present are excessively minute ; other and richer sources were sought for. It was found that such varied organic materials as yeast, moulds, maize germ oil and urine contained considerable amounts of auxin or of substances which had the same effect on growth. From urine were first isolated two organic compounds, called auxin *a* and auxin *b* and it was possible to determine their chemical structure. Later, a further compound, differing in its chemical nature from the first two, was obtained and identified as indole acetic acid. This has been of special importance, for it can be synthesised rather readily, and is available in pure form in quantities and at a price adequate for research. But, although it is now known that indole acetic acid does occur in the higher plant, it is highly probable that the auxin of the oat coleoptile is identical with auxin *a*. The activity of all these substances is astonishingly great. 1 mg. of indole acetic acid is sufficient to produce a 10 degree curvature in 25,000,000 oat seedlings.

We may return for a moment to the phototropic curvature. It has been possible to show, using the methods invented by Went, that the shoot of a plant bends towards the light from two causes ; (1) part of the auxin on the illuminated side is inactivated, and (2) part is deflected, so that it passes down the dark side. The amount of auxin on the dark side is therefore relatively greater and that side grows more rapidly so that a curvature towards the light is produced. It may be noted that a similar explanation applies to geotropism. If a shoot is laid horizontal the auxin accumulates on the lower side, which grows faster and causes the shoot to bend up. The downward growth of a root is also due to an auxin effect, but here the relation to growth is more complex and is not yet fully cleared up.

The tropistic movements thus explained are secondary effects; the fundamental importance of auxin is that it is essential to growth. Growth is a very complex chain of events, but in the plant one most important link is the extension of the cellulose wall of the cell, a stretching which is accompanied by an incorporation of new cellulose molecules in the structure. The actual stretching is caused by the internal turgor pressure of the cell and it is either in rendering the wall more plastic or in the process of incorporation of new molecules that the auxin acts. We do not yet know the precise mode of action.

But this, though fundamental, is only one of the activities shown by auxin. Of others the best known is that on root production. If the base of a cutting is immersed in a very weak solution of indole acetic acid for a few hours the subsequent production of roots may be very strikingly increased. This has great practical importance. It has an additional interest from the scientific point of view, for the production of roots on a cutting begins, not with the expansion of the walls of existing cells, but by the formation of new cells and a new growing point by cell division. From the practical side a good deal of work has been done with rather disappointing results so far. It has been found that, in those plants which root fairly readily, the production of roots is accelerated and intensified by auxin applications. But the plants which root with difficulty are not much affected. These are, of course, the most interesting to the horticulturist, whose work would be much easier if artificial means of improving the striking of difficult plants could be found. The work on these problems was interrupted by the war and no doubt fresh ground will be broken when it is taken up again and the conditions of rooting are made clearer.

Already another approach to the problem has been made. The organic chemist is able to start with a substance like indole acetic acid and synthesize others related to it. For example, it is possible to substitute butyric, propionic and other organic acids for the original acetic acid. Or he may use a ring other than the indole ring and make substances like naphthalene acetic acid or naphthoxy-acetic acid. Some of these materials are at least as powerful as, and in some relations more powerful than, indole acetic acid. This has been a line of work which has produced important results in other connections, as in the development of the sulphonamide drugs and the organic arsenicals.

Another growth effect concerned with the formation of new cells, which we now know to be dependent on auxin, is the activity of the cambium. Cambium is a sheet of cells lying between the wood and the rind of woody plants. The divisions which take place in it provide the cells which form new wood to the inside and new bast to the outside and so bring about the growth in thickness of tree and shrub. In herbaceous plants cambium also functions, though its effect is less conspicuous. The cambium is dormant in winter and breaks into activity in spring, the season in which the young cells it forms are so flushed with sap that they rupture easily and so allow the rind to be stripped from the wood. This activity starts with a renewed supply of auxin, formed in the buds as they begin to open. It is just below the buds that cambial activity starts, and, as the supply of auxin passes further down branch and trunk, so the activity of the cambium spreads downwards. It may reach the roots only some weeks after it has started in the branch tips.

Even more striking is the relation of auxin to another case of fresh growth—that which leads to the development of seed and fruit. In the higher plants pollen is transferred from stamen to stigma ; the pollen tube grows down and penetrates the ovule in the ovary ; the male nucleus, thus carried to the ovule, fuses with the egg cell there and fertilisation has taken place. From the fertilised egg cell develops the embryo which is found in the seed. The remainder of the ovular tissues, as the embryo develops, enter into growth and form the seed coats and endospermic food stores. The first impulse to this renewed growth may be given by auxin brought in by the pollen tube, for pollen is known to be a rich source. But, as soon as the embryo starts development, it produces auxin itself and so, as it were, catalyses its own growth. It also starts the growth of the ovary, which in turn develops into the fruit. This is the normal course, but we know of various cultivated plants which form fruits without pollination, fertilisation or seed production. Examples of such *parthenocarpic* fruits are the seedless raisins and oranges, the pineapple, the banana and some cucumbers. In these the ovary itself seems to produce enough auxin to start its development into a fruit. It has now been found possible to produce parthenocarpic, and seedless, fruits in a variety of plants by the application of auxin to their stigmas and ovaries. The tomato can be made to bear a full and normal crop of fruits, without pollination and containing no seeds, by applying a very fine spray containing minute quantities of synthetic auxins. This may make possible the solution of a problem of considerable economic importance. Many fruit trees lose a large number of immature fruits by fruit drop. A late frost may cause the whole of a crop of young fruits to fall. The whole of the apple crop in our district was destroyed in this way in 1945. The application of auxin may save the crop in such circumstances. It has already been found possible to prevent the fall of the young fruits, though not yet to make them swell and develop normally. Intensive work is in progress on this problem.

The formation of flowering shoots is controlled by many factors external and internal. It is now certain that an idea, put forward by the German physiologist, Sachs,[2] nearly a century ago, is correct and that the plant produces hormone-like substances which are essential for flower production. These are not the same as the auxins which control growth, and we do not yet know their chemical nature, for they have not been isolated. The proof of their existence is indirect and has been obtained through certain curious relations of flowering to light and temperature. There are plants, like the strawberry, which flower only in the long days of early summer, that is, with long periods of illumination : and others, such as the chrysanthemum, which flower only in the shorter days of autumn. There are plants which flower only after they have been subjected to a period of low temperature in their youth ; such are the winter cereals, which are sown in autumn and pass the winter as seedlings. There is much evidence that such effects are due to the flowering hormones being formed only in the appropriate conditions of light or temperature. We can note only one point. If a plant which has been kept in the appropriate conditions is suitably grafted on one which has not, the influence—the hormone—is transmitted to the latter and it will then flower.

The higher plant, in contrast with the higher animal, is a very loosely knit organism. Large parts may be cut away without any serious damage being done, and pieces cut off may be rooted and give rise to new plants. But it nevertheless is an integrated organism developing harmoniously, producing a characteristic form, and flowering and fruiting in due course. How is this integration brought about ? Partly by external circumstance and partly by internal events. The latter may be nutritional, through the supply of water or of food substances to one part or another. But internal regulation through the auxins and other hormones is of vital importance. Auxin produced in one part affects the growth, whether by extension or by cell division, in others. The flowering hormones, probably formed in the leaves, determine the onset of reproduction. One further instance of this internal regulation may be given. A most surprising discovery was made when it was found that the same substance which stimulates growth in most conditions, can also inhibit it. In the angle between each leaf and the stem which bears it, lies a bud. This bud may develop into a branch or it may lie dormant. In trees and shrubs most buds lie dormant : if they all grew out the result would be a thicket and not the shapely tree we know. It is the *presence* of the main growing tip which keeps the buds dormant. If the apex of the shoot is cut off the buds lower down, or some of them, grow out. This is, of course, how the gardener produces a bushy plant. But if the tip is cut off, and indole acetic acid is applied to the wounded surface, the buds remain dormant. It is the flow of auxin from the tip which prevents growth from starting. Various theories have been put forward to explain this surprising fact but none that is satisfactory. The effect is certain, but the explanation is yet to be given.

The work surveyed has taken place in the last twenty years and it is evident that a whole new field in the physiology of growth has been opened up. It is interesting to look at the way in which this development has taken place. It started with a problem, the mechanism of the phototropic movement, of great interest to the scientist, and indeed to anyone attracted by the phenomena of plant behaviour. It was not a problem the solution of which might be thought likely to give results of any practical import: yet practical applications have emerged. It is an example of the way in which the establishment of some fundamental principle is the preliminary to developments which could not have been foreseen. Among these may be developments of practical importance. The deliberate, frontal attack on such a problem as the production of parthenocarpic fruit could have been planned; but whatever resources had been used there could have been no guarantee of success. The unplanned, or better, natural advance of science, in seeking primary knowledge of growth processes, made the way clear for the solution of all sorts of derived problems. In the following of these new lines the planning of research is in place.

REFERENCES.

[1] Went, F. W., *Rec. trav. bot. néerl.*, 1928, vol. xxv.

[2] Sachs, *Arb. Bot. Inst. Würzburg*, 1880, 1882, vols. i. and ii.

